# Expression profiling of small cellular samples in cancer: less is more

**DOI:** 10.1038/sj.bjc.6601668

**Published:** 2004-02-24

**Authors:** J G Glanzer, J H Eberwine

**Affiliations:** 1 Department of Pharmacology, University of Pennsylvania Medical Center, Philadelphia, PA 19104-6058, USA

## Abstract

Expression profiling of tumours from cancer patients has uncovered several genes that are critically important in the progression of a normal cell to an oncogenic phenotype. Leading the way in these discoveries is the use of microarrays, a technology that is currently in transition from basic science applications to use in the clinic. Microarrays can determine the global gene regulation of an individual cancer, which may be useful in formulating an individualised therapy for the patient. Currently, cells used in breast cancer microarray studies often come from either homogenous cultures or heterogeneous biopsy samples. Both cell sources are at a disadvantage in determining the most accurate gene profile of cancer, which often consists of multiple subspecies of cancerous cells within a background of normal cells. Therefore, acquisition of small, but highly specific biopsies for analysis may be required for an accurate expression analysis of the disease. Amplification methods, such as polymerase chain reaction (PCR) and amplified antisense RNA (aRNA) amplification, have been used to amplify the mRNA signal from very small samples, which can then be used for microarray analysis. In this study, we describe the acquisition, amplification, and analysis of very small samples (<10 000 cells) for expression analysis and demonstrate that the ultimate resolution of cancer expression analysis, one cell, is both feasible and practical.

Many human diseases are intricately linked to aberrant changes in gene expression. Cells with arguably the most profound genetic reprogramming are found in cancer, a disease that accounts for nearly one-quarter of all deaths in the United States (2003). Fortunately, advances in cancer drug development and other therapeutics have reduced mortality rates in several types of cancer.

While the mortality rates for several types of cancer have been reduced, little change has been seen in the mortality rate of breast cancer, a statistic that is complicated by the fact that even though improved therapies have been developed, an everincreasing caseload is being generated from an increased lifespan. In response to these high mortality rates, public and private funding entities have made breast cancer research a high priority, which has accelerated the use of newly developed diagnostic technologies for early detection to be used in the breast cancer. As an example of how small sample mRNA analysis may help in the evaluation and treatment of cancer, we will concentrate our discussion upon breast cancer, for which there has been significant molecular analysis over the last decade.

## BREAST CANCER

One of the striking aspects of breast cancer is the variability in its phenotypic manifestation across different patients (Murphy *et al*, 1995). Doubling rates of growth of breast cancer tumours in patients can range from 2 weeks to 5 years. The progression of these tumours into metastatic disease is unpredictable as well, attesting to the heterogeneity of breast cancer from patient to patient. Although breast cancers can be classified by several indices including tumour size, metastasis, etc., the current standard for determining treatment of metastatic breast cancer is lymph node involvement and the expression of three specific genes: the oestrogen receptor, the progesterone receptor and the HER-2/*neu* oncogene (Bast *et al*, 2001). The identification of these genes as important markers for breast cancer is the result of many years of classical expression profiling, where only a few genes could be monitored at one time. These methods have also uncovered several other genes (*CAE, Ki-67, Ca 15-3, Ca 27, Ca 29, cathespin*) that may eventually be used in breast cancer diagnosis and treatment (Bast *et al*, 2001). However, to uncover the totality of genes that contribute to the breast cancer phenotype, future directions will require a genome-wide approach where all genes in a cancer can be assayed simultaneously. Total gene surveillance can be accomplished by the use of microarrays.

### MICROARRAY TECHNOLOGY

Microarray-based expression profile technology is based on hybridisation methods first employed nearly 30 years ago (Southern *et al*, 1999). RNA from cells are reverse transcribed into cDNAs, which are then fluorescently or radioactively labelled and used to probe a predetermined set of DNAs. The greater the hybridisation signal from probe-DNA binding, the higher the concentration of the RNA within the original sample. Currently, upwards of 35 000 genes can be analysed at one time on a microarray.

Initial expression profiling efforts used large amounts of cells (>10^6^ cells), often harvested from large tumours or cell lines (Forozan *et al*, 2000). Although cancers arise from a single cellular event, analysis of dissected tumours has revealed a high degree of heterogeneity within the cell mass (Wild *et al*, 2000). Cells isolated from the periphery of the tumour can have mRNA expression patterns distinct from cells located deep within the tumour. Cells that metastasise elsewhere in the body can acquire several genotypical and phenotypical differences from those of the primary cancer, which may allow these cells to react differently to therapy than those at the primary site (Nishizuka *et al*, 2002). Therefore, harvesting of and analysis of gross quantities of tumour for expression analysis may not give an accurate assessment of the cancer needed for determining which therapeutic intervention to use.

The large amount of RNA needed for microarray analysis (2–10 *μ*g) can also be problematic. For instance, the use of mammography and early detection campaigns has resulted in early detection of smaller tumours that can be sampled for expression analysis. Fine-needle aspiration (FNA), a method for obtaining small amounts of tumour cells (<100 000) for histological identification of cancer has become the standard for tumour sampling (Krawczyk *et al*, 2000). However, the amount of RNA that can be harvested from a FNA biopsy (∼2 *μ*g) is at the lower limit for use in microarray studies without the use of amplification methods (Assersohn *et al*, 2002; Symmans *et al*, 2003). Fine-needle aspiration samples also contain a high percentage of noncancerous cells (20–60%), making a true representation of a cancer expression profile difficult (Assersohn *et al*, 2002; Symmans *et al*, 2003).

**Figure 1:**
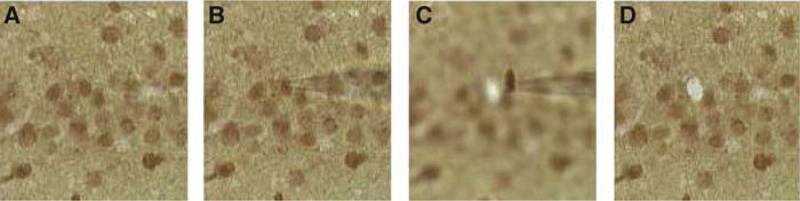
mRNA harvesting for single cells. These panels show the progression for harvesting of RNA from a single cell in a fixed tissue section. Initially, *in situ* transcription is performed on the tissue with the amplification primer followed by immunohistochemistry to identify cells expressing a particular antigen. The immunopositive cells are imaged under a microscope (**A**) and a patch pipette is apposed to an individual cell (**B**) and the material aspirated into the pipette (**C**) leaving a hole where the cell was removed (**D**). The cDNA is removed from the isolated tissue and processed through the aRNA amplification procedure. These photos were taken by Paolo Marciano for studies detailing changes in gene expression associated with apoptosis.

Cancer-specific cellular samples for microarray analysis can be obtained through microdissection of tumour sections (Wild *et al*, 2000; Zhu *et al*, 2003). Cells obtained from biopsies can be mounted and isolated using laser capture microdissection (LCM), then placed into a microcentrifuge tube for cDNA synthesis. Samples containing as few as 1–10 cells can be isolated in this manner, and subsequently used in microarray experiments (Kamme *et al*, 2003; Seshi *et al*, 2003). Alternatively, similar samples can also be obtained through manual microdissection, where single-cell isolation can be accomplished using micromanipulators and pulled glass micropipettes as scalpels (Eberwine *et al*, 1992; O'Dell *et al*, 1998). Once separated from the cell matrix, the cell body is aspirated into the micropipette and placed into a sample tube for analysis. It is important to note that prior to dissection with this procedure, the mRNA is converted into cDNA using an oligonucleotide primer (Tecott *et al*, 1988). This is one distinction between LCM isolated mRNAs that are generally nonfixed and the manual microdissection of fixed tissues. The harvesting of a single cell from a fixed tissue section is shown in Figure 1

. This method should be quite effective in the isolation of pure cancer cell samples from FNAs. Fine-needle aspiration samples can be plated and fixed onto glass coverslips. After immunostaining, individual positive-stained cells can easily be aspirated. Single-cell dissection can be further optimised by using a microchisel. The vibrating blade allows efficient cutting of the cell body from the cell matrix without disrupting neighbouring cells. The microchisel can also be used in the dissection of highly elastic nonfixed tissue, raising the possibility of using live cells for expression profiling. Live cells may be more attractive for expression profiling of small samples, as the mRNA is not affected by fixatives that can crosslink the RNA to proteins and other molecules, resulting in lower RNA yields (author's observation). This procedure on live cells has permitted subregions of live cells to be harvested and assayed for mRNA abundances using macroarrays (Miyashiro *et al*, 1994; Crino and Eberwine, 1996).

## MRNA AMPLIFICATION

**Figure 2:**
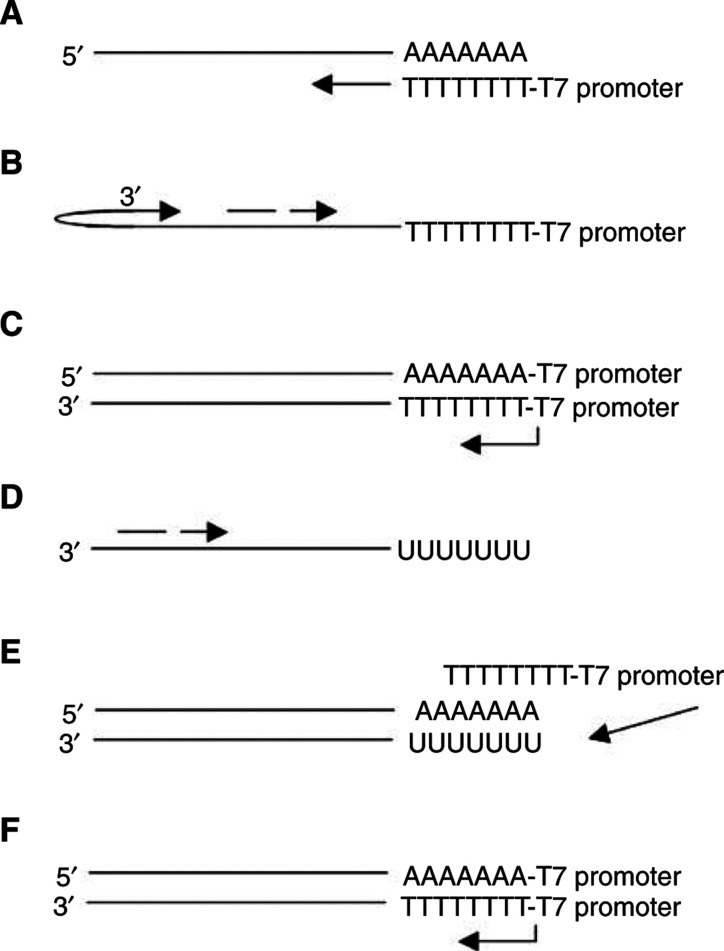
First and second rounds of the aRNA procedure. (**A**) mRNA is reverse transcribed using polyT-T7 primer. (**B**) Second-strand synthesis primed by hair-pining or random primers. (**C**) T7 RNA polymerase-mediated transcription, creating amplified antisense RNA. (**D**) Reverse transcription of antisense RNA using random hexamers. (**E**) Second-strand synthesis using polyT-T7 primer. (**F**) T7 RNA polymerase-mediated transcription, again creating antisense RNA. Further rounds of amplification are performed by repeated steps (**D**) through (**F**).

As it becomes necessary to sample smaller amounts of cells for microarray analysis, it becomes clear that an amplification of the cDNA signal is needed. Polymerase chain reaction, a powerful exponential amplification procedure, can be used for quantitative gene-specific amplification of individual cDNAs (Stirling, 2003). However, PCR is limited in its ability to globally amplify the entire transcriptome of a cell, as PCR amplifies long, GC-rich sequences with less efficiency than short, AT-rich sequences, resulting in skewed amplification levels from gene to gene that are carried over into each successive (>30) round (Polz and Cavanaugh, 1998). Owing to of these limitations, most microarray amplification methods make use of a linear-based amplification method using T7 RNA polymerase, resulting in an amplified RNA (aRNA) (Figure 2

) (Van Gelder *et al*, 1990). In this strategy, polyA RNA is primed for cDNA synthesis by a polyT oligonucleotide containing the 17 bp sequence for the T7 RNA polymerase promoter. After second-strand synthesis is completed, the template is then transcribed by a highly concentrated T7 polymerase, resulting in a ∼2000-fold amplification of antisense RNA that can be used for hybridisation analysis (Phillips and Eberwine, 1996).

Currently, most microarray studies that utilise the aRNA synthesis protocol require one amplification round, allowing as little as 10 ng or 1000 cells to be used for the initial input (Sotiriou *et al*, 2002; Yunes *et al*, 2003). However, smaller amounts of cellular material require a second round of amplification. A second (and any subsequent) round requires the use of random primers in reverse transcribing the output aRNA from the preceding round. The polyT-T7 oligonucleotide is then used to prime second-strand synthesis. The resulting template is again transcribed by T7 RNA polymerase, creating a second amplification of aRNA. As each round of aRNA synthesis can amplify the template 2000-fold, a second round will result in a >10^6^-fold amplification. At this level of amplification, the transcriptome of a single cell can be analysed (Eberwine *et al*, 1992; Cheetham *et al*, 1997; Kamme *et al*, 2003). Subsequent cycles of aRNA amplification have been successfully performed and the products are used as probes on microarrays (Xiang *et al*, 2003). The only limitation for extra rounds of amplification arises from the use of random primers, which results in a 30% shortening of the transcript after each round (personal observation). Therefore, the aRNA method will have a 3′ bias. This bias will have only a slight effect on the hybridisation of cDNA-based arrays that utilize the entire gene sequence. Oligonucleotide-based microarrays are often designed using 3′ sequences of genes, and therefore may not be affected by this 3′-end bias (Luzzi *et al*, 2003). This 3′-end bias may be eliminated through the use of random dodecamer-T7 oligonucleotide primers that will prime cDNA synthesis along the length of the mRNA, not simply at the 3′-end. This comes at the price of generating smaller cDNAs and having a large amount of ribosomal RNA amplified as part of the probe.

## USE OF SINGLE-CELL PROFILING IN BREAST CANCER

The heterogeneity and unpredictable behaviour of breast cancer strongly encourage a closer look into the cast of ‘cellular characters’ that are responsible for the invasive and proliferative nature of this disease. Single-cell profiling would be helpful in characterising cells that have specific roles in the cancerous phenotype.

Invasive tumour cells can release factors that allow them to pass through several layers of epithelium (Nozaki *et al*, 2000). Since cancer cells that border normal tissue are in a great minority to those cells within the tumour mass, whole tumour expression profiling would not easily allow the identification of genes associated with this invasive cell subtype. Indeed, differences in gene expression have been seen within different areas of breast cancer carcinomas (Zhu *et al*, 2003). The identification of new gene markers at the tumour interface through single-cell profiling could allow for a more precise measurement of the invasiveness of the tumour, and may one day help in the decision-making process for effective therapeutic action.

In a similar fashion, tumour cells that border sites of angiogenesis may express genes that promote the formation of blood vessels to the tumour. Through single-cell profiling, specific genes that are found differentially regulated in these cells relative to noncancerous host cells may be used as drug targets for combating cancer-induced angiogenesis.

Probably one of the most powerful aspects of single-cell profiling would be the tracking of a cancer at the cellular level as the patient undergoes treatment. Active cancers often release millions of living cells into the bloodstream daily, where they can be identified in a blood sample though fluorescence-activated cell sorting or plating procedures, then analysed for mRNA expression (Kim *et al*, 2003; Weigelt *et al*, 2003; Zabaglo *et al*, 2003). As the patient goes through days or weeks of treatment, expression profiles from individual cancer cells isolated from blood would give an indication of which particular subtype of cancer cell is most prevalent in the body. Data from these experiments could prove powerful in determining the mechanisms of selection of a resistant tumour subtype for a particular therapy, and may be beneficial in developing therapeutic strategies based upon the expression profile of the initial circulating cells.

Finally, a major advantage of using a multigenic approach to the analysis of cancer-associated gene expression in single cells is that the alteration of abundances of multiple mRNAs suggests that the protein levels for the corresponding mRNAs may likewise be altered. Further, there is the implication that if mRNA abundances are altered, then the abundance of corresponding functional proteins may also be altered. While this is not necessarily the case, it is reasonable to follow up microarray data by determining protein abundances (Zhang *et al*, 2001) and protein functional data. In this manner, insight into combinations of proteins that are altered as a result of cancer may be determined. This information may permit the identification of multiple drugs that act on different proteins, which when used in combination may prove to be an effective cancer therapeutic. Such combinatorial therapies that selectively impact a cancerous cell would be difficult to develop without single-cell gene and protein expression data.

## CONCLUSION

The increased longevity of the Western population has come with the price of increasing numbers of cancer cases. As we try to develop better chemotherapeutics for combating this disease, we must first elucidate the mechanisms that contribute to the phenotype. Given the heterogeneity of inter- and intracellular phenotypes associated with any cancer, it is reasonable to suggest that cancer may be understood as the sum of its parts. By identifying the minute differences in mRNA and protein abundances between cancer cell subtypes in patients, we may develop combinatorial therapeutics that will combat the cancer with a higher rate of success than current single-target drugs.
